# Hypoxia Tolerance Declines with Age in the Absence of Methionine Sulfoxide Reductase (MSR) in *Drosophila melanogaster*

**DOI:** 10.3390/antiox10071135

**Published:** 2021-07-17

**Authors:** Nirthieca Suthakaran, Sanjana Chandran, Michael Iacobelli, David Binninger

**Affiliations:** Department of Biological Sciences, Charles E Schmidt College of Science, Florida Atlantic University, Boca Raton, FL 33431, USA; nsuthaka@my.fau.edu (N.S.); schandran2017@fau.edu (S.C.); iacobellim2015@fau.edu (M.I.)

**Keywords:** aging, repair, methionine sulfoxide reductase, *Drosophila*, hypoxia, oxidoreductases, oxidative stress, physiological function

## Abstract

Unlike the mammalian brain, *Drosophila melanogaster* can tolerate several hours of hypoxia without any tissue injury by entering a protective coma known as spreading depression. However, when oxygen is reintroduced, there is an increased production of reactive oxygen species (ROS) that causes oxidative damage. Methionine sulfoxide reductase (MSR) acts to restore functionality to oxidized methionine residues. In the present study, we have characterized in vivo effects of MSR deficiency on hypoxia tolerance throughout the lifespan of *Drosophila*. Flies subjected to sudden hypoxia that lacked MSR activity exhibited a longer recovery time and a reduced ability to survive hypoxic/re-oxygenation stress as they approached senescence. However, when hypoxia was induced slowly, MSR deficient flies recovered significantly quicker throughout their entire adult lifespan. In addition, the wildtype and MSR deficient flies had nearly 100% survival rates throughout their lifespan. Neuroprotective signaling mediated by decreased apoptotic pathway activation, as well as gene reprogramming and metabolic downregulation are possible reasons for why MSR deficient flies have faster recovery time and a higher survival rate upon slow induction of spreading depression. Our data are the first to suggest important roles of MSR and longevity pathways in hypoxia tolerance exhibited by *Drosophila*.

## 1. Introduction

Tolerance to a diminished level of oxygen (hypoxia) is a complex process that leads to a variety of responses by different organisms. The mammalian brain only tolerates a few minutes of severe hypoxia before it causes irreversible cell damage [1]. In contrast, *Drosophila melanogaster* has evolved a mechanism through which it can tolerate several hours of hypoxia without significant tissue injury. It does this by entering a protective coma known as spreading depression. Currently, there is limited knowledge of how the conserved cellular signaling pathways may modulate the susceptibility and vulnerability of the brain to spreading depolarization (SD) [2]. However, when oxygen is reintroduced by reperfusion, there is an increased production of reactive oxygen species (ROS).

ROS are a group of highly reactive molecules that contain oxygen including radicals, molecules with unpaired valence electrons. Examples of ROS are superoxide (O_2_^−^), hydrogen peroxide (H_2_O_2_), and the hydroxyl radical (OH) [3]. The accumulation of ROS is an unavoidable byproduct of cellular respiration, primarily within the mitochondrial electron transport chain. Superoxide is formed from the partial reduction of oxygen due to electron leakage at complex I and III of the electron transport chain [4]. ROS can also be produced through external sources such as UV light and radiation. Some ROS molecules lead to oxidative stress by oxidizing macromolecules and other cellular components, causing functionality loss [4]. For example, ROS oxidizes the sulfur atom of methionine residues, oxidizing it to methionine sulfoxide (met-(o)) and often leading to the loss of protein function [5,6]. However, at low levels, certain ROS molecules, such as hydrogen peroxide, can also have a positive function since they regulate core metabolic pathways and play a role in signaling [4,7]. To prevent oxidative damage, cells can destroy the ROS molecules before they harm cellular components with the help of specific enzymes. Superoxide dismutase (SOD) is a well-studied example. SOD, which is present in the mitochondria and cytoplasm, converts the superoxide ion into hydrogen peroxide. Glutathione peroxidase (GPX) then reduces the hydrogen peroxide to water [4].

Oxidative damage can be reversed. The sulfur-containing amino acid, methionine, present in peptides and proteins play a role in antioxidant defense, catalysis, protein structure, and redox sensing and regulation [8]. The interconversion of methionine and methionine sulfoxide (met-(o)), in vivo, involves enzyme-catalyzed redox reactions where methionine forms met-(o) by adding oxygen to its sulfur atom. The chiral center at the sulfur atom brings in two epimers of met-(o), met-R-(o) and met-S-(o). The reaction can be catalyzed by methionine sulfoxide reductase [9]. Methionine sulfoxide reductase (MSR) represents a family of enzymes that have the role of reversing oxidative damage by reducing met-(o) back to its original form. Evidence for MSR activity was described by Weissbach, Brot and colleagues when they observed that oxidation of methionine residues that inactivated ribosomal protein L12 in *Escherichia coli* [10] could be restored by MSRA [11]. There are two forms of MSR designated MSRA and MSRB, which are responsible for the stereospecific reduction of met-(o). MSRA reduces the S enantiomer (met-S-(o)) while MSRB reduces the R enantiomer (met-R-(o)). The thioredoxin (Trx) and glutathione (GSH) systems are thiol-dependent antioxidant mechanisms in cells that are part of DNA synthesis and repair by acting as an electron donor for ribonucleotide reductase (RNR) and methionine sulfoxide reduction by giving electrons to MSR [12,13,14]. Mammals including humans have one *MSRA* gene and three distinct *MSRB* genes (*MSRB1*, *MSRB2*, *MSRB3*). *Drosophila* also has a single *MSRA* gene, but it only has one *MSRB* gene.

According to the free radical theory of aging, the accumulation of ROS and other free radicals play a significant role in aging by causing oxidative damage [15]. Previous studies have detected increased ROS production in aged tissues, emphasizing the link between oxidative damage and aging [16]. In addition, met-(o) levels in proteins have been shown to increase with age in several aging models, such as replicative senescence and erythrocyte aging. Decreased levels of MSRA have been found in aged mouse tissues, and MSRA and MSRB were found to be downregulated during replicative senescence of human WI-38 fibroblasts [17]. Therefore, it is evident that there is a relationship between oxidative stress, the level of MSR activity, and aging. However, further studies are necessary to fully understand the role of MSR in aging and the onset of neurodegenerative diseases.

This study examines the effects of MSR in aging for *Drosophila melanogaster*. More specifically, it investigates the effect of MSR-deficiency on hypoxia tolerance throughout the lifespan of the adult *Drosophila*.

## 2. Materials and Methods

### 2.1. Fly Stocks

*Drosophila* stocks were maintained on standard cornmeal agar medium (Genesee Scientific, El Cajon, CA, USA) at 25 °C with a 12-h light/dark cycle. The WT60 strain is homozygous wild-type for both the *MSRA* and *MSRB* loci (*MSRA*^+/+^*MSRB*^+/+^). The AB46 strain is fully deficient for any MSR activity due to homozygous null alleles for both the *MSRA* and *MSRB* genes (*MSRA*^∆/∆^MSR*B*^∆/∆^). The generation and characterization of these genetic lines has been described [18].

### 2.2. Hypoxia Treatment

Male flies were maintained with 50 animals per vial. All experiments were done between 12:00–5:00 p.m. to minimize changes in behavior affected by circadian rhythms. Chronic hypoxia was induced and maintained by continuous displacement of air in the hypoxia chamber (63.5 cm long × 33.7 cm wide × 43.8 cm high) with nitrogen gas (100%) at the calculated flow rate using Weymouth’s formula [19]. The rapid nitrogen flow rate was calculated as 2.0 L/s., the moderate flow rate was 1.4 L/s and the slow flow rate was 25 mL/s. An outlet in the chamber prevented any pressure changes within the chamber. A Pasco PASPort oxygen sensor (Pasco; Roseville, CA, USA) was used to confirm that the concentration of oxygen inside the chamber was between 0–5% regardless of the nitrogen flow rate. The hypoxic conditions were maintained for the indicated time and nitrogen flow rate. Flies were then returned to standard atmosphere and monitored for recovery from the spreading depression coma.

### 2.3. Monitoring Fly Movement

Animal movement was monitored using a Drosophila Activity Monitor (DAM; TriKinetics Inc., Waltham, MA USA) which uses 32 glass tubes in an 8 × 4 array. One fly is placed in each tube which has small holes to allow free exchange of gasses. An infrared detector monitors the movement of the fly, recording each time the animal moves through the beam of the detector. Each trial began with an acclimation period of 10 min before data recording commenced. The onset of the spreading depression coma was measured as the time to the first cessation of movement detected by the DAM monitoring system. After the indicated time of hypoxic/reoxygenation stress, flies were returned to normal atmosphere. The time to recovery was scored as the first movement of the animal detected by the DAM movement detection system. Flies that remained comatose after five hours of recovery were marked as failing to survive the hypoxia.

### 2.4. Statistical Analyses

Data were analyzed using Prism statistical software (GraphPad Software, San Diego, CA, USA). The sample number (*n*) in each trial represents the number of flies, one in each glass tube, whose individual movements were monitored by the DAM movement detection system. Data points falling outside (outliers) the interquartile range (IQR) were not used in determining the average, standard error of the mean (SEM), sample number (*n*) and statistical significance.

## 3. Results

### 3.1. Characterization and Age-Grouping of Wild-Type and MSR-Deficient Strains

Complete loss of function (null) alleles of *MSRA* and *MSRB* were created by imprecise *p*-element transposon excision [18]. Briefly, the *MSRA* null allele is a 1.5 kb deletion that starts 300 bp upstream of the transcription start site and extends into Exon 2. The entire 5′ UTR and a portion of the open reading frame have been removed. The *MSRB* null allele is a 2.5 kb deletion that starts 364 bp upstream of the transcription start site and terminates 2163 bp into the transcribed region. The loss of these genomic sequences removes the first three exons that includes a part of the open reading frame.

Strains that are homozygous wild-type for both *MSR* loci, homozygous for the *MSRA* deletion, homozygous for the *MSRB* deletion and MSR-deficient lines due to being homozygous for both deletions were used for this study. Experiments involving one hour of exposure to hypoxia were used for all four strains. However, no significant and reproducible differences between the wild-type strain and the strains that were homozygous for only one mutant locus were observed. Therefore, all the reported experiments only involve wild-type (WT60) and MSR-deficient (AB46) strains.

The absence of any functional *MSR* loci leads to nearly a 50% shorter lifespan compared to the wildtype [18]. One copy of the wild-type allele of either MSRA or MSRB was found to be sufficient to almost completely rescue the lifespan of the MSR double-deletion mutant to nearly that of the wildtype line (*p* < 0.001). The MSR double-deletion flies do not survive past 40–45 days [18]. Preliminary experiments showed that there were age-related differences in the response of the two strains to hypoxia. Animals were examined at a young age, middle age, and old age. Adjustments were made to the time period for each stage in their lifespan to account for the significantly shorter lifespan of the MSR-deficient line (Table 1).

### 3.2. Rapid Induction of the Spreading Depression Coma

A flow rate of 2.0 L/s nitrogen gas induced a spreading depression coma in both wild-type and MSR-deficient flies in less than one minute. Onset of the protective coma occurred too quickly to determine whether there was a difference between the two strains.

**Figure 1:**
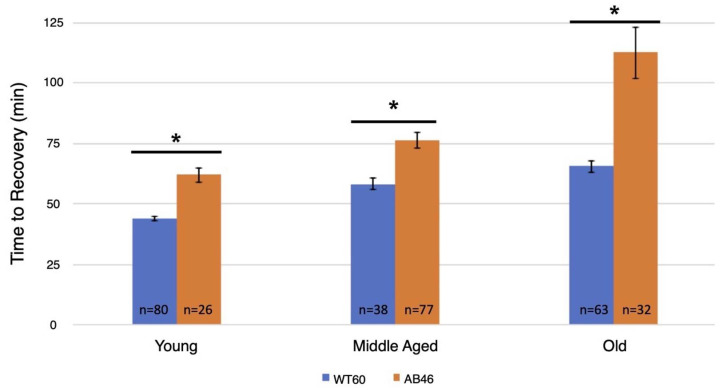
Recovery after one hour hypoxia under rapid induction of spreading depression. Wild-type (WT60) and MSR-deficient (AB46) animals were exposed to one hour of hypoxia using a nitrogen flow rate of 2.0 L/s. After one hour of hypoxia, the animals were returned to normoxic conditions and allowed to recover from the spreading depression coma. Recovery was marked as the time to the first movement detected by the Drosophila Activity Monitor. The number of individual animals (*n*) used for each age-group and genotype is shown on the graph. Error bars are the SEM. Significance is indicated by an asterisk where *p* < 0.0001 using an unpaired *t*-test.

After one hour of acute hypoxia due to continuous air displacement with nitrogen, the animals were returned to normoxic (standard atmosphere) conditions and allowed to recover. Recovery was scored as the first movement detected by the infrared detector of the Drosophila Activity Monitor (see methods). There was an age-dependent effect in the recovery times for both wild-type and the MSR-deficient flies with an increase in recovery time as the animals aged (Figure 1). However, the MSR-deficient animals took significantly longer (*t*-test; *p* < 0.0001) compared to wild-type to recover at every age through their entire lifespan. This difference was most notable among the old animals where the wildtype took an average of 65.6 min to recover, but the MSR-deficient animals took 72% longer with an average of 112.8 min (*p* < 0.0001).

### 3.3. Moderate and Slow Induction of Hypoxic Coma

**Figure 2:**
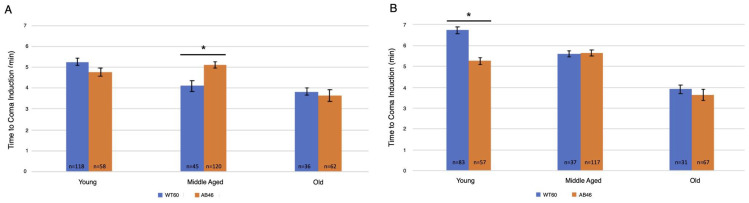
Moderate and slow induction of spreading depression. Wild-type (WT60) and MSR-deficient (AB46) animals were exposed to one hour of hypoxia. A nitrogen gas flowrate of 1.4 L/s was used for a moderate rate of induction (**A**) while a flow rate of 25 mL/s was used for a slow rate of induction (**B**). Onset of the coma was scored when the IR detector no longer recorded movement. The number of individual animals (*n*) used for each age-group and genotype is shown on the graph. Error bars are the SEM. Significance is indicated by an asterisk where *p* < 0.0001 using an unpaired *t*-test.

Two slower flow rates of nitrogen were used to extend the time to onset of the spread-ing depression coma to better determine whether there was an effect due to the absence of MSR activity. Onset of the coma was determined by the time required to no longer detect movement by the IR sensor of the Drosophila Activity Monitor. Overall, there was an age-dependent decrease in the time to induce the coma for both the moderate (Figure 2A) and slow (Figure 2B) flow rates of nitrogen gas. Interestingly, significant differences in the time for coma induction only occurred among middle age flies using the moderate flow rate and young flies using the slow flow rate. Wildtype middle age flies took an average of 4.1 min (*n* = 45) whereas the MSR-deficient flies took 24% longer with an average of 5.1 min (*n* = 120; *p* < 0.0001). Under the slow induction of spreading depression, wild-type flies took an average of 6.75 min (*n* = 83) which was 28% longer than the MSR-deficient flies which took an average of 5.3 min (*n* = 57; *p* < 0.0001).

### 3.4. Recovery Following Moderate and Slow Induction of Hypoxia

**Figure 3:**
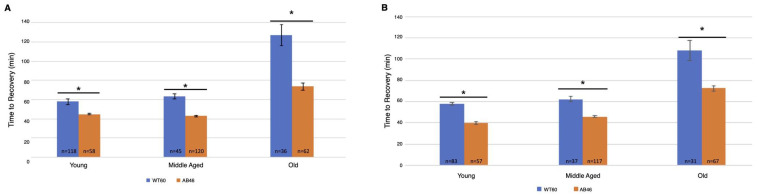
Recovery after one hour hypoxia under moderate or slow induction of spreading depression. Wild-type (WT60) and MSR-deficient (AB46) animals were exposed to one hour of hypoxia using a moderate nitrogen flow rate of 1.4 L/s (**A**) or a slow flow rate of 25 mL/s (**B**). After one hour of hypoxia, the animals were returned to standard atmosphere and allowed to recover, their activity was scored as the first movement detected by the Drosophila Activity Monitor. The number of individual animals (*n*) used for each age-group and genotype is shown on the graph. Error bars are the SEM. Significance is indicated by an asterisk where *p* < 0.0001 using an unpaired *t*-test.

The animals were returned to a normal atmosphere after one hour of hypoxic exposure to nitrogen gas at the indicated flow rate. Recovery was monitored as the first movement detected by the IR sensor as described above. There was an overall increase in recovery time for both wild-type and MSR-deficient animals as they aged. Unexpectedly, we found that the MSR-deficient animals recovered significantly faster than the wild-type at every age group for both the moderate (Figure 3A) and slow (Figure 3B) nitrogen flow rates. The difference was most pronounced in the old animals. When the coma was induced under a moderate nitrogen flow rate, old wild-type flies took an average of 127.2 min which was 73% longer than the average of 73.6 min required for recovery of the old MSR-deficient animals (*p* < 0.0001). The old wild-type animals recovered more quickly when the slow flow rate of nitrogen was used whereas recovery of the old MSR-deficient animals was nearly the same as those under a moderate nitrogen flow rate (73.6 min vs. 72.4 min). The wild-type flies still recovered more slowly with an average time of 108.0 min which was 49% longer than the average of 72.4 min required by the old MSR-deficient flies (*p* < 0.0001).

### 3.5. Survival Following One Hour of Hypoxia

**Figure 4:**
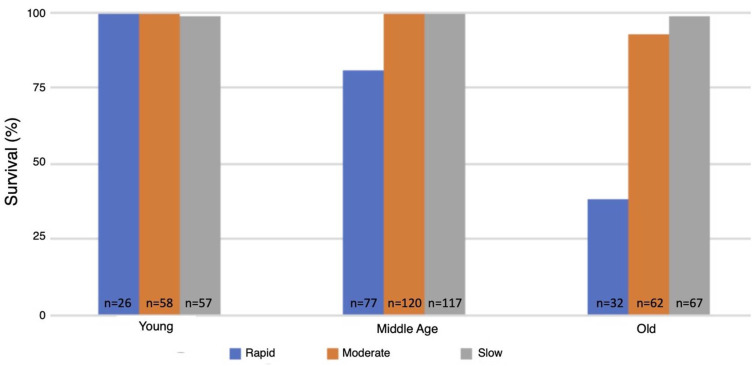
Survival following hypoxia of the MSR-deficient animals. The MSR-deficient (AB46) flies were subjected to 60 min hypoxia using the indicated flow rate of nitrogen. The flies were then returned to standard atmosphere and monitored for recovery from the coma as described in the legend of Figure 2. Animals that did not recover movement within 5 h were scored as not surviving.

The flow rate used for inducing the spreading depression coma had a major effect on the survival of animals as they aged. Survival of the wild-type strain was 99–100% at all ages tested and under all three modes for inducing spreading depression (data not shown). In contrast, survival of the MSR-deficient strain (AB46) as the animals aged was strongly dependent on mode of coma induction. The young MSR-deficient animals had a 99–100% survival irrespective of the nitrogen flow rate, which was like the survival success of the wild-type strain (Figure 4). At middle-age, the MSR-deficient had a 93–99% survival when the moderate or slow flow rate was used but survival declined to 79% using the rapid nitrogen flow rate (Figure 4). The effect of the nitrogen flow rate on survival was most pronounced among the old MSR-deficient flies. Nearly all the animals (99%) survived with the slow nitrogen flow rate. Even at the moderate nitrogen flow rate, the survival rate was 93%. However, under the rapid flow rate, where the MSR-deficient animals took 72% longer than the wildtype to recover from the hypoxia (Figure 1), only 38% of the animals survived.

### 3.6. Effects of Prolonged Hypoxia on Recovery and Survival

All the previous experiments used one hour of hypoxic exposure. However, wild-type *Drosophila* can survive hours of chronic hypoxia [20,21]. Therefore, we examined the effect of three hours and six hours of hypoxia on the recovery and survival of the MSR-deficient flies. For these experiments, the slowest flow rate (25 mL/s) was used since the recovery and survival of the MSR-deficient flies was most robust.

**Figure 5:**
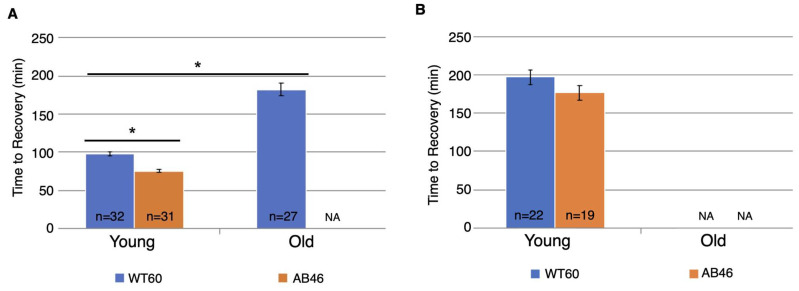
Recovery after prolonged hypoxia. Wild-type (WT60) and MSR-deficient (AB46) animals were exposed to three hours (**A**) or six hours (**B**) hypoxia using the slow nitrogen flow rate (25 mL/s). After the indicated time of hypoxia, the animals were returned to normal atmosphere and allowed to recover from the spreading depression coma. Recovery was scored as the first movement detected by the Drosophila Activity Monitor. The number of individual animals (*n*) used for each age-group and genotype is shown on the graph. NA indicates that none of the test animals survived the hypoxic treatment. Error bars are the SEM. Significance is indicated by an asterisk where *p* < 0.0001 using an unpaired *t*-test.

Young MSR-deficient flies recovered significantly faster than the wild-type after three hours of hypoxia, using the slow flow rate of nitrogen (Figure 5A). Young wild-type flies took 98.2 min to recover which was 31% longer than the 75.5 min for recovery of the young MSR-deficient animals (*p* < 0.0001). After six hours of hypoxia (Figure 5B), the wild-type flies took twice as long to recover (197.0 min) compared to three hours of hypoxia. The MSR-deficient flies also took longer to recover (176.5 min) after the two additional hours of hypoxia (compare Figure 3 to Figure 5). While the MSR-deficient strains recovered faster than the wild-type after six hours of hypoxia, the difference was not significant (*p* = 0.08).

**Figure 6:**
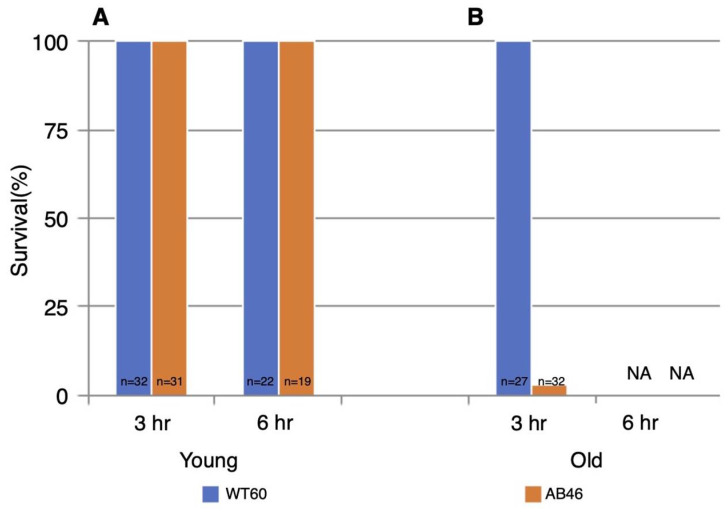
Survival following prolonged hypoxia. Young flies (**A**) or old flies (**B**) were subjected to either 3 h or 6 h of anoxia using a 25 mL/s flow rate of nitrogen. Flies were then returned to standard atmosphere and monitored for recovery from the spreading depression coma as described in the legend of Figure 2. Animals that did not recover movement within 5 h were scored as not surviving the hypoxia. NA indicates that none of the animals survived.

All the young test animals of both genotypes survived both 3 h and 6 h of hypoxia (Figure 6). However, there was a striking effect of age. All the old wildtype flies survived 3 h of hypoxia whereas only 3% old MSR-deficient flies (1 of 32 animals) survived. In sharp contrast to the young animals, none of the old flies of either genotype survived six hours of hypoxia.

## 4. Discussion

Although the role of MSR in oxidative stress has been extensively studied, the investigation into how the absence of MSR activity affects hypoxia tolerance in *Drosophila melanogaster* is a relatively new area of exploration. Fruit flies were discovered to be tolerant to acute hypoxia (0 mm Hg O_2_) in the early 1990s, where they survived in oxygen depleted environments for several hours without any evidence of injury [1]. The disruption of oxygen homeostasis is a major factor for many disease etiologies and pathobiology [22]. The low oxygen conditions (hypoxia) used in this study is a prominent clinical problem associated with many diseases such as ischemic heart disease, cerebral ischemia (stroke), pulmonary hypertension, diabetes complication, high altitude illness, and cardio-respiratory disorders (bronchopulmonary dysplasia and obstructive sleep apnea). The evolutionary conservation of genetic and signaling pathways from *Drosophila* to mammals allows for it to be an ideal model system to investigate the genetic basis of hypoxia tolerance [22]. Fruit flies enter a protective coma called spreading depression, which allows survival to prolonged periods of hypoxia, by suppressing their overall metabolic rate to prevent cellular injury during reoxygenation [23]. Insect spreading depolarization provides new insight for mammalian spreading depolarization due to its association with stress-induced neural shutdown and energy conservation response [24]. Hypoxia/reoxygenation induces cellular injury through the promotion of oxidative stress, which was the impetus to quantify recovery during these conditions. ROS cause oxidative damage to amino acids, lipids, nucleic acids, and play a crucial role in aging and senescence [25]. The MSR system protects vital cell constituents from oxidative stress and redox regulation of signaling pathways [26].

The loss of all known MSR activity has been reported in bacteria [27] and yeast [28], although these organisms are not as developmentally complex as *Drosophila*. The correlation between the loss of MSRA activity and reduction in longevity were documented in yeast [28], *C. elegans* [29] and mice [30]. However, a previous study on an *MSRA* knockout mouse did not find an effect on lifespan [31]. In our lab, *MSRA* and *MSRB* gene deletions were created through imprecise excision of *p*-element transposons located in each gene, leading to the establishment of *Drosophila* as an in vivo animal model to lack any known MSR activity. We have previously found a significantly shorter lifespan in the *MSR* double mutant with nearly full restoration of normal lifespan in the presence of a single wild-type allele of either *MSRA* or *MSRB* [18]. Our previous experiments failed to identify a significant phenotype in *Drosophila* lacking just one of the two *MSR* genes. In fact, there is a significantly longer third instar with larvae growing at a slower rate and adult flies having a shortened lifespan in the absence of any MSR activity (*MSRA*^∆/∆^*MSRB*^∆/∆^) [18]. Similarly, we found key behavioral differences on the effects of hypoxia under conditions that vary the rate which the spreading depression coma is induced, as well as the length of hypoxia/reoxygenation stress.

The time required for flies to cease movement due to the protective coma induced by hypoxia can be varied by altering the rate at which the chamber becomes hypoxic (i.e., by controlling the flow rate of the nitrogen gas). In our study, the survival of flies was dependent on neural function because of the differences in onset of spreading depression. After sudden exposure to hypoxia, the *MSR* double mutant flies took significantly longer to recover compared to the wildtype flies throughout the flies’ entire lifespan (Figure 1). More interestingly, the MSR-deficient flies showed a markedly reduced ability to survive the hypoxic/reoxygenation stress as they approached senescence with only 38% surviving among the flies that were 40–45 days old (Figure 4). In contrast, the wildtype strains had nearly 100% survival of the hypoxia throughout their entire lifespan including the period of senescence (60–65 days old) (data not shown). According to the oxidative stress theory of aging, as animals age, an increase in ROS and oxidative stress plays a role in governing lifespan. Previous research suggesting that the accumulation of oxidative damage is part of the aging process is an outdated approach, suggested as far back as 1954. Aging is rather a genetically programmed series of events that increases fitness of each species [32]. For example, the oxidation of methionine residues activates the key enzyme involved in calcium signaling pathway, multifunctional calcium/calmodulin-dependent protein kinase II (CaMKII). Mice that were MSRA^−/−^ displayed exaggerated CaMKII oxidation and myocardial apoptosis, leading to impairment in cardiac function and increased mortality under myocardial infarction. These previous in vivo studies confirmed a physiologically regulatory role for methionine oxidation of CAMKII in cardiomyocytes. In fact, overexpression of the MSRA gene predominantly in the nervous system was found to extend *Drosophila* lifespan. MSRA transgenic animals were more resistant to paraquat-induced oxidative stress with a remarkable delay in the onset of senescence-induced decline in activity as well as reproductive capacity [33]. MSRA could be also involved in regulating target protein function and/or expression in ROS-mediated signal transduction. Changes in gene expression, including those of oxidative stress-response genes, were previously seen when MSRA is overexpressed [34]. Our experiments are the first evidence of an age-dependent effect of MSR deficiency in both recovery and survival from hypoxic/reoxygenation stress.

Being that we measured hypoxia tolerance through percent survival and/or recovery time, we were curious to see whether this would be altered if spreading depression was induced more slowly. The onset of spreading depression was not affected by the lack of MSR activity but there was an age-dependent decrease in the time to induce the coma for both the moderate (Figure 2A) and slow (Figure 2B) flow rates. However, upon reoxygenation, the MSR-deficient flies displayed a stark contrast to previous experiments and recovered faster than wildtype flies throughout their entire lifespan (Figure 3). More interestingly, the survival of the MSR-deficient flies improved dramatically and was nearly 100% throughout the entire lifespan (Figure 4). Our results suggest slow induction of spreading depression may allow for ischemic preconditioning to commence. There has been interest in understanding the nature of ischemia-reperfusion (IR) injury and therapy development to prevent its effects [35,36]. The first few minutes of reperfusion initiates long-term tissue damage and dysfunction [36]. Early studies revealed a role for mitochondrial activity in early IR injury, where the reperfusion through a burst of reactive oxygen species (ROS) production is from the mitochondria [37,38]. Ischemia can alter the abundance of mitochondrial metabolites that act as electron stores and CoQ reductants upon reperfusion [36,39,40,41]. Succinate was a common signature of ischemia throughout metabolically diverse tissues, where it was found to accumulate more than any other mitochondrial metabolite [39]. Numerous studies have showed an increase in succinate during ischemia or anoxia [40,41]. Thus, reducing ischemic succinate accumulation or slowing succinate metabolism at reperfusion is known to be cardioprotective against IR injury [42]. This may be why MSR-deficient flies recover faster and have markedly improved survival when the induction of hypoxia occurs more slowly [43].

The hypoxia tolerance in fruit flies permits survival of extended hypoxia without neuronal deficit, due to the protective coma they entered during hypoxia [44]. Our next set of experiments focused on studying hypoxic tolerance upon prolonged hypoxia (3 and 6 h) and the slow induction of spreading depression. Young animals had 100% survival for both the wild type and MSR-deficient strains up to six hours of slow induction of hypoxia (Figure 6). At old age, 100% of the wildtype flies survived three hours of hypoxia although none of the animals survived six hours of hypoxia (Figure 6B). In sharp contrast, only 3% (1 of 32 flies) of the old MSR-deficient flies survived the three hours of hypoxia (Figure 6B). There is a clear age-dependent decline in the ability to survive prolonged hypoxia in the absence of MSR. Young MSR-deficient flies continued to recover significantly faster than wildtype animals upon prolonged hypoxia, while old MSR-deficient flies did not recover at all (Figure 5). The underlying mechanism may be associated with the relationship between the production and depletion of cellular energy during spreading depression. During hypoxia, the metabolic rate is known to significantly decrease to allow *Drosophila* to preserve cellular ATP while also decreasing its total production [20,45,46,47]. When flies start to recover upon reoxygenation, there is less available ATP to restore metabolic deficits due to the ATP depletion during the period of hypoxia. Overall, survival is compromised [45,48]. In addition, ATP depletion is known to lead to failure of the Na^+^/K^+^ ATPase, which results in dysregulation of ionic homeostasis, protein unfolding and subsequently protein aggregation [49]. Numerous reports in the literature reflect similar trends of a strong inverse correlation between increased stress duration and decreased survival probability, possibly due to deficiency in ATP production and the inability of the fly to compensate for ATP consumption [20,47].

## 5. Conclusions

The results obtained from this study demonstrate that MSRA and MSRB play an age-dependent role in protection against oxidative stress throughout the lifespan of *D. melanogaster*. MSRA and MSRB are known to behave as antioxidants to reduce methionine sulfoxide (nonfunctional form of methionine from ROS oxidation) back to the functional form of methionine [50]. The original expectation was that MSR-deficient flies would have a compromised ability to tolerate hypoxia. The results of experiments using the MSR-deficient flies suggest new lines of inquiry involving ischemic preconditioning and longevity pathways. These results support previous studies that suggest the activation of protective mechanisms to defend against oxidative stress, essentially leading us to a better understanding how these *MSR* genes affect aging. Our studies offer possible insight into hypoxic-like conditions in humans, such as stroke, that may ultimately contribute to better drug design or other treatments.

## Figures and Tables

**Table 1:** Categorization of age groups.

Age Group	Wild-Type	MSR-Deficient
Young	20–25 Days Old	5–10 Days Old
Middle Age	40–45 Days Old	30–35 Days Old
Old	60–65 Days Old	40–45 Days Old

MSR-deficient animals (AB46) have a markedly shorter lifespan. The ages selected to identity young, middle-age and old adult flies for each genotype are based on a previous study, as described in the text.

## Data Availability

Data is contained within the article.
